# Analysis of porcine adipose tissue transcriptome reveals differences in de novo fatty acid synthesis in pigs with divergent muscle fatty acid composition

**DOI:** 10.1186/1471-2164-14-843

**Published:** 2013-12-01

**Authors:** Jordi Corominas, Yuliaxis Ramayo-Caldas, Anna Puig-Oliveras, Jordi Estellé, Anna Castelló, Estefania Alves, Ramona N Pena, Maria Ballester, Josep M Folch

**Affiliations:** 1 Centre de Recerca en Agrigenòmica (Consorci CSIC-IRTA-UAB-UB), Edifici CRAG, Campus UAB, Bellaterra, 08193 Barcelona, Spain; 2 Departament de Ciència Animal i dels Aliments, Facultat de Veterinària, Campus UAB, Bellaterra, 08193 Barcelona, Spain; 3 INRA, UMR 1313 Génétique Animale et Biologie Intégrative (GABI), Equipe Génétique Immunité Santé, Jouy-en-Josas F-78352 France; 4 AgroParisTech, UMR 1313 GABI, Jouy-en-Josas F-78352 France; 5 CEA, DSV/iRCM/SREIT/LREG, Jouy-en-Josas F-78352 France; 6 Departamento de Mejora Genética Animal, INIA, Ctra. de la Coruña km. 7, 28040 Madrid, Spain; 7 Genètica i Millora Animal, IRTA Lleida, 25198 Lleida, Spain

**Keywords:** RNA-Seq, Transcriptome, Adipose tissue, Pork, *de novo* lipogenesis

## Abstract

**Background:**

In pigs, adipose tissue is one of the principal organs involved in the regulation of lipid metabolism. It is particularly involved in the overall fatty acid synthesis with consequences in other lipid-target organs such as muscles and the liver. With this in mind, we have used massive, parallel high-throughput sequencing technologies to characterize the porcine adipose tissue transcriptome architecture in six Iberian x Landrace crossbred pigs showing extreme phenotypes for intramuscular fatty acid composition (three per group).

**Results:**

High-throughput RNA sequencing was used to generate a whole characterization of adipose tissue (backfat) transcriptome. A total of 4,130 putative unannotated protein-coding sequences were identified in the 20% of reads which mapped in intergenic regions. Furthermore, 36% of the unmapped reads were represented by interspersed repeats, SINEs being the most abundant elements. Differential expression analyses identified 396 candidate genes among divergent animals for intramuscular fatty acid composition. Sixty-two percent of these genes (247/396) presented higher expression in the group of pigs with higher content of intramuscular SFA and MUFA, while the remaining 149 showed higher expression in the group with higher content of PUFA. Pathway analysis related these genes to biological functions and canonical pathways controlling lipid and fatty acid metabolisms. In concordance with the phenotypic classification of animals, the major metabolic pathway differentially modulated between groups was *de novo* lipogenesis, the group with more PUFA being the one that showed lower expression of lipogenic genes.

**Conclusions:**

These results will help in the identification of genetic variants at *loci* that affect fatty acid composition traits. The implications of these results range from the improvement of porcine meat quality traits to the application of the pig as an animal model of human metabolic diseases.

## Background

The pig (*Sus scrofa*) is one of the most important livestock animals due to its economic importance in the alimentary industry, but it is also an interesting biomedical model for human diseases [1]. Over the last few decades, genetic selection in commercial pig breeds has greatly improved meat-production efficiency at the expense of reducing the sensorial and technological properties of the meat. These changes are mainly caused by the reduction in intramuscular fat (IMF) content and alterations in fatty acid (FA) composition, both critical for various meat quality attributes such as muscle color, firmness, water-holding capacity and also important nutritional aspects [2]. In this regard, FA composition of food has also become a critical aspect in human nutrition: a high consumption of SFA has been associated with obesity, high plasma cholesterol and cardiovascular diseases [3,4], while replacing SFA with MUFA or PUFA decreases serum LDL cholesterol and total cholesterol, reducing the risk of coronary heart disease [5,6].

Factors other than dietary intake have been less characterized in relation with tissue lipid composition. These include the role of candidate-gene genotypes in lipid and FA metabolism [7-12]. In this context, studies in pigs can have a dual purpose: first, to study the genetics of food, i.e., how the genotype of the animal influences the FA content and profile of meat and, secondly, as an animal model for nutrigenomic studies or for human metabolic diseases.

The liver, adipose tissue and skeletal muscle are the principal organs involved in the regulation of lipid metabolism. The adipose tissue is an organ which is responsible for energy storage in the form of lipids and, in pigs, is the major source of circulating free FAs (FFA) [13]. It also acts as a major endocrine organ, producing adipocytokines like TNFα, peptide hormones such as leptin, adiponectin, estrogen and resistin and lipid hormones (lipokines) such as palmitoleate, all of which are involved in the maintenance of metabolic homeostasis [14,15]. Furthermore, pig adipose tissue has a greater contribution to overall FA synthesis than does the liver [13]. Thus, the characterization of the transcriptome landscape of this organ may be relevant for the improvement of pork nutritional quality.

The development of next-generation sequencing (NGS) methods has provided new tools for both transcriptome characterization and gene-expression profiling. The RNA-Seq technique is based on sequencing the poly-A RNA fraction and allows for characterizing isoforms from known genes or discovering novel, predicted coding genes [16]. To-date, the number of RNA-Seq analyses in livestock is still scarce, some recent reports have focused on the study of organs [17,18], animal products such as milk [19,20] or embryos [20]. Thus, in 2011, Esteve-Codina *et al.* compared the pig gonads of two individuals from two breeds (Iberian and Large White). The same year, Chen *et al.* (2011) analyzed the transcriptome of three pig tissues (liver, *longissimus dorsi* muscle and abdominal fat) in two full-sib F_2_ females with extreme phenotypes in growth and fatness (White Duroc × Erhualian). Furthermore, a liver RNA-Seq study was performed using four animals from genetically different porcine breeds (Berkshire, Duroc, Landrace and Yorkshire) [21].

In a previous work of our group, the livers of ten Iberian × Landrace backcrossed pigs classified in two phenotypically-extreme groups for intramuscular FA composition (five per group), were analyzed using RNA-Seq [22]. This study identified 55 genes differentially expressed in the liver that may play a crucial role in muscle lipid composition. Nevertheless, muscle lipids are derived not only from the liver (mostly dietary lipids), but also from adipose tissue (mostly *de novo* lipogenesis) [15,23]. Therefore, the aim of the present study is to investigate the contribution of backfat transcriptome to the FA content and profile of intramuscular fat in pigs. The two main goals of our study are: (i) the identification of genes and pathways differentially expressed in the backfat of Iberian × Landrace crossbred pigs (BC1_LD) showing extreme phenotypes for intramuscular FA composition, and (ii) to describe transposable elements and new putative protein-coding genes in the transcriptome of pig backfat. Combining the new adipose tissue transcriptome data with the already available liver transcriptome information will allow us to study the expression of genes regulating the overall lipid metabolism in pigs.

## Results and discussion

### Phenotypic variations between extreme groups

In a previous work by our group, animals from an Iberian × Landrace backcross (BC1_LD) were analyzed with a Principal Component Analysis (PCA) to describe the phenotypic variation of traits related to carcass quality and intramuscular FA composition [22]. The score information of the first principal component in PCA was used to classify the BC1_LD animals into two groups High (H) and Low (L) and the hepatic transcriptome from the most extreme females (five per group) was evaluated using an RNA-Seq approach [22]. In the present study, a total of six females (three per group) were selected for RNA sequencing of their backfat tissue. Pedigree information was used to avoid the selection of sibs in the same group. When phenotypic means between groups were compared, 50% of the traits showed significant statistical differences (14/28) (Additional file 1: Table S1). In summary, the H group showed higher levels of intramuscular polyunsaturated fatty acids (PUFA), and group L showed higher levels of saturated fatty acids (SFA) and monounsaturated fatty acids (MUFA) (Additional file 1: Table S1). The lack of statistical significance of palmitoleic (C16:1 n-7) and heptadecenoic (C17:1) acids content, in comparison with Ramayo-Caldas *et al.* (2012), may be explained by the lower sample size. Results obtained are in agreement with the breed effect on backfat FA composition, showing similarities between L-group animals and Iberian pigs, which had higher percentages of C16:0 and MUFA (particularly C18:1) and a lower content of PUFA than did commercial breeds [24-26]. Meanwhile, animals of the H group had higher percentages of PUFA, as observed in Landrace animals [26]. Most importantly, the two groups of pigs did not differ in either IMF content or backfat thickness. This is relevant, as the two groups include pigs with similar abilities to deposit fat in muscle and backfat, but they incorporate FA with different elongation and desaturation indexes.

### Characterization of pig adipose tissue transcriptome

Sequencing yielded around 236 M of 75-bp paired-end reads and approximately 84% of reads were mapped against the reference pig genome assembly Sscrofa10.2 using Tophat software. Percentages of mapped reads observed per individual ranged from 80% to 87%, and were equally distributed in the H and L groups. These values were higher than were the percentages reported in previous porcine transcriptome studies: 71.4%-77.8% [22], 61.4%-65.6% [27], 66.7% [28] and 54% [21]. The IntersectBed tool from BEDtools was employed to calculate the proportion of mapped reads annotated in exons, introns and intergenic regions. The highest percentages of reads were mapped to exons (66%-71%), while 19%-20% fell in intergenic regions, and the lowest percentages were located in introns (10%-14%) (Table 1). The proportion of reads mapped to exons was slightly higher than were the values reported by Ramayo-Caldas *et al.* (2012) and Chen *et al.* (2012), due likely to the better pig genome assembly (Sscrofa10.2) and annotation version employed in our work. Nevertheless, the current gene annotation still needs to be improved in order to determine the function of approximately 20% of reads, which mapped in intergenic regions. Finally, transcripts generated from assembling the short reads by cufflinks fell mostly into annotated exons (42%-47%). The remaining reads were classified into the following categories: intron retention events (7%-12%), intergenic transcripts (17%-20%), potentially novel isoforms of genes (19%-21%), pre-mRNA molecules (3%-4%) and polymerase run-on fragments (2%-3%) (Additional file 1: Table S2). The percentage of intergenic transcripts represented the third category in read abundance, which is relevant for detecting putative coding transcripts or new transposable elements not described in the current version of the pig genome.

**Table 1:** Summary of mapped reads

**Animal**	**Group**	**Number of reads**	**% Exons**	**% Introns**	**% Intergenic**
**BC1**	L	78,149,606	71	10	19
**BC2**	L	93,736,238	71	10	19
**BC3**	L	66,287,842	67	13	20
**BC4**	H	89,531,208	69	12	20
**BC5**	H	62,002,574	66	14	20
**BC6**	H	83,335,152	69	12	19

L = low; H = high.

### Exploring for novel coding transcripts and transposable elements in the adipose tissue transcriptome

Transcript annotation performed with cufflinks showed a mean value of 10,862 total unknown intergenic transcripts (Additional file 1: Table S2). This value doubles the number of intergenic transcripts detected in previous studies [22,28]. To determine which of these transcripts encoded a protein, Augustus software [29] was used and, as was expected, the total amount of predicted proteins was also higher, as compared to previous studies: 4,130 predicted proteins against 326 [22] and 714 [28]. Our analysis showed an improvement in the sequence length and sequence coverage. Furthermore, the conservative approach used in [22], in which only those transcripts expressed in at least four of the five animals of each group were considered, could aid to explain the differences obtained (4,130 vs. 326). BLASTP analysis was performed to compare the putative proteins predicted by Augustus against the predicted proteins reported by Ramayo-Caldas *et al.* (2012) in the liver and Esteve-Codina *et al.* (2011) in gonads. A total of 269 new putative proteins fitted in with those previously described: 93 putative proteins were expressed in liver transcriptome (34%) and 211 were expressed in gonad transcriptome (78%). Additionally, a functional annotation was performed using BLAST2GO software. BLASTP analysis of the 4,130 predicted proteins revealed that 2,100 proteins (50.8%) displayed significant similarity with existing protein sequences (the top hit species was *Sus scrofa*, 59.1%). These proteins corresponded to: 16 novel computationally predicted proteins and 1,108 known human proteins, 361 novel and 598 known bovine proteins and 997 novel and 599 known porcine proteins. Hence, the number of novel predicted proteins was lower in better-described genomes (human and bovine). From the 2,100 predicted proteins, only 1,226 were functionally annotated with at least one gene ontology (GO) term. At the third GO level, the most represented biological terms were ‘primary metabolic process’ (8% of predicted proteins), ‘cellular metabolic process’ (8%) and ‘regulation of biological process’ (7%). According to the molecular function, ‘protein binding’ (25%), ‘ion binding’ (17%) and ‘nucleic acid binding’ (16%) were the most represented categories. In the cellular compartments category, ‘cell part’ (29%) was the most represented term, followed by ‘membrane-bounded organelle’ (20%) and ‘organelle part’ (16%) (data not shown). Furthermore, the main metabolic pathways represented were purine metabolism (49 sequences), pyrimidine metabolism (11), phosphatidylinositol signaling system (11) and inositol phosphate metabolism (10); these and other pathways observed are shown in Additional file 2: Table S3.

Repetitive elements (RE) were identified in the adipose tissue transcriptome using the RepeatMasker software. The total interspersed repeats represented 36% of the intergenic transcripts (Additional file 3: Table S4), a percentage higher than those observed in previous porcine transcriptome analyses: 5.8%-7.3% [22] and 7.3% [28]. As described above, the differences obtained could be explained by the assembly used for each analysis. The description of these regions has been improved in the current assembly (Sscrofa10.2), as demonstrated in the results observed. The major part of the RE were classified as SINEs (n = 92,007), representing 13.96% of the transcriptome sequenced. The second group was LINEs (n = 43,428), but these RE constitute a larger part of the transcriptome than do SINEs due to the bigger size of LINEs (15.88% of adipose tissue transcriptome). The remaining RE were classified as: LTR elements (4.04%), DNA elements (2.06%) and unclassified RE (0.02%).

### Gene expression analysis

Counting the reads mapped in each gene, around 15,747 annotated genes were expressed in adipose tissue with similar amounts between groups (L = 15,608-16,001, H = 15,433-15,834). Taking into account only those genes with a minimum mean of 20 reads per gene in at least one of the extreme groups, 13,086 expressed genes were selected. Gene-expression distribution was similar in both groups, classifying 1% of the selected genes between 0–20 mapped reads; 27%-28% among 20–200 mapped reads; most genes (46%-47%) had between 200–2,000 mapped reads; 23% among 2,000-20,000 mapped reads, and the remaining genes (2%) more than 20,000 mapped reads (Additional file 4: Figure S1). Mean gene-expression levels were highly correlated between groups (r = 0.98 between H and L groups), indicating that most genes were similarly expressed in both groups. Five of the six individuals were also assayed with the Gene-Chip® Porcine microarray (*Affymetrix*; Santa Clara, CA) to analyze the gene expression of 20,201 *Sus scrofa* genes. After signal normalization, correlation between the expression data obtained by microarrays and RNA-Seq was calculated. All animals showed a high Spearman correlation (r = 0.65-0.68) (Additional file 5: Figure S2) in accordance with previous studies of the porcine transcriptome [22,28,30], confirming the reproducibility of the data. Genes with intermediate expression values had a higher correlation between technologies than did genes with low or high expression values. This same pattern had already been observed in previous studies, and it is explained by the higher dynamic range of RNA-Seq analyses [30,31]. Finally, the top 100 expressed genes showed an over-representation in biological gene ontologies related to hormone-sensitive lipase-mediated triacylglycerol hydrolysis, lipid digestion, mobilization and transport, pyruvate metabolism and biosynthesis of unsaturated FAs. Additionally, key regulatory pathways were also detected: the PPAR signaling pathway, which is important for the induction of pre-adipocyte differentiation and FA storage [32], or the ChREBP transcription factor, which has emerged as a major mediator of glucose action on lipogenic gene expression and as a key regulator of lipid synthesis [33].

### Differential gene expression among animals with extreme phenotypes of intramuscular FA composition

**Figure 1:**
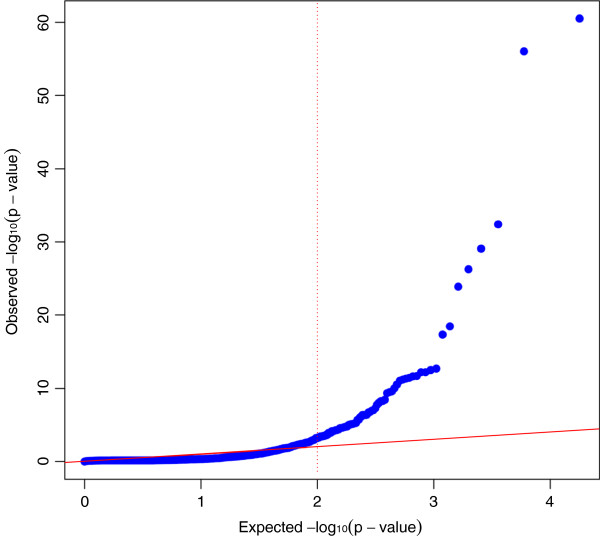
**Q-Q plot representing the distribution of the p-value.** The red line represents the expected distribution of the p-value, while the blue trend represents the distribution observed. X-axis values are Expected –log_10_ (p-value) and y-axis are the Observed –log_10_ (p-value).

**Figure 2:**
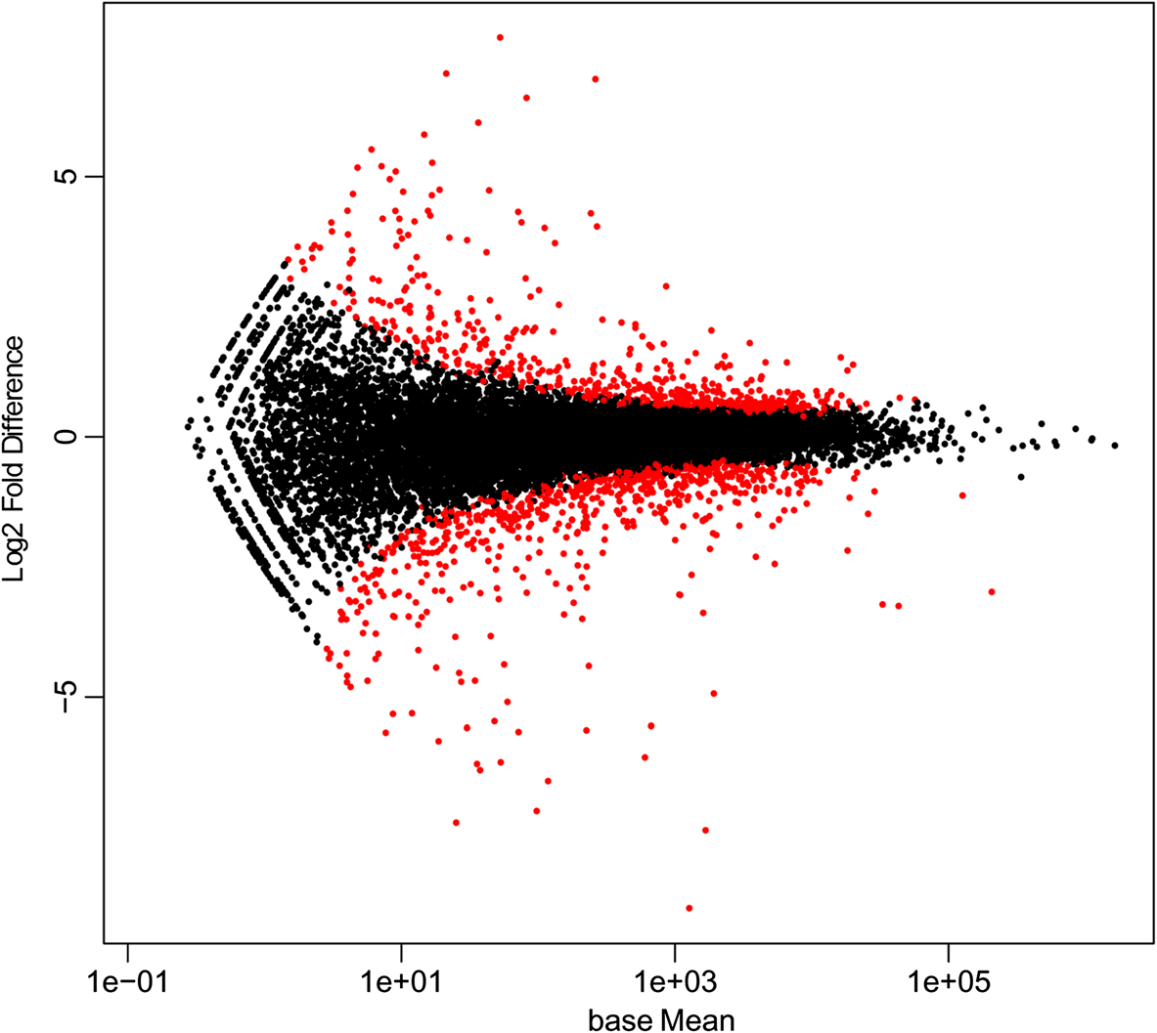
**Representation of the 396 differentially expressed genes (in red) with fold difference ≥ 1.2 and p-value ≤ 0.01.** X-axis values are base mean-expression values and y-axis values are the log2 (fold difference).

Biological functions over-represented in the differentially expressed genes are candidate functions to explain the variation in intramuscular FA composition among the analyzed animals. Gene-expression data from each group was compared using DESeq software, which allowed for the detection of differentially expressed genes between groups. The data were filtered, discarding those genes with a mean of less than 20 reads mapped in one of the extreme groups. Finally, a total of 13,086 genes were used to perform the differential expression analysis using a standard cut-off of: |fold difference| ≥ 1.2, a p-value ≤ 0.01 and a q-value ≤ 0.1 (Figure 1). DESeq software identified a total of 396 differentially expressed genes (Figure 2), from which 247 were up-regulated in the L group and the remaining 149 genes were down-regulated in the same group (Additional file 6: Table S5). It is noteworthy that nine of the differentially expressed genes in adipose tissue (*IVD*, *CRABP2*, *SLC2A12*, *AACS*, *RBP1*, *ACADL*, *APOB* and *THEM5*) have been previously reported as being located in genomics regions associated with the profile of intramuscular FA composition in a GWAS study in the same animal population (Table 2) [34]. Therefore, these genes should be considered as interesting candidate genes for pig meat quality traits. Nevertheless, future studies are needed to determine the causative effect of each gene, due to RNA-Seq analyses not allowing us to distinguish the causal genes from those genes that are consequently altered by the gene expression of other genes or by phenotypic differences.

**Table 2:** **Differentially expressed genes associated with intramuscular FA composition in a genome-wide association study in the same population**[34]

**Ensembl Gene ID**	**Gene name**	**Counts L group**	**Counts H group**	**p-value**	**Fold difference**
**ENSSSCG00000004172**	SLC2A12	316.27	124.56	5.2E-03	−2.54
**ENSSSCG00000004774**	IVD	10,800.27	4,058.75	3.21E-06	−2.66
**ENSSSCG00000008596**	APOB	26.38	106.03	1.3E-03	4.02
**ENSSSCG00000006472**	CRABP2	1,215.36	463.39	1.9E-04	−2.62
**ENSSSCG00000006614**	THEM5	23.62	152.32	8.4E-05	6.45
**ENSSSCG00000011664**	RBP1	861.18	403.95	4.1E-03	−2.13
**ENSSSCG00000009755**	AACS	5,287.19	2,303.84	2.7E-05	−2.29
**ENSSSCG00000016156**	ACADL	4,533.01	2,366.33	1.6E-03	−1.92

L = low; H = high.

**Figure 3:**
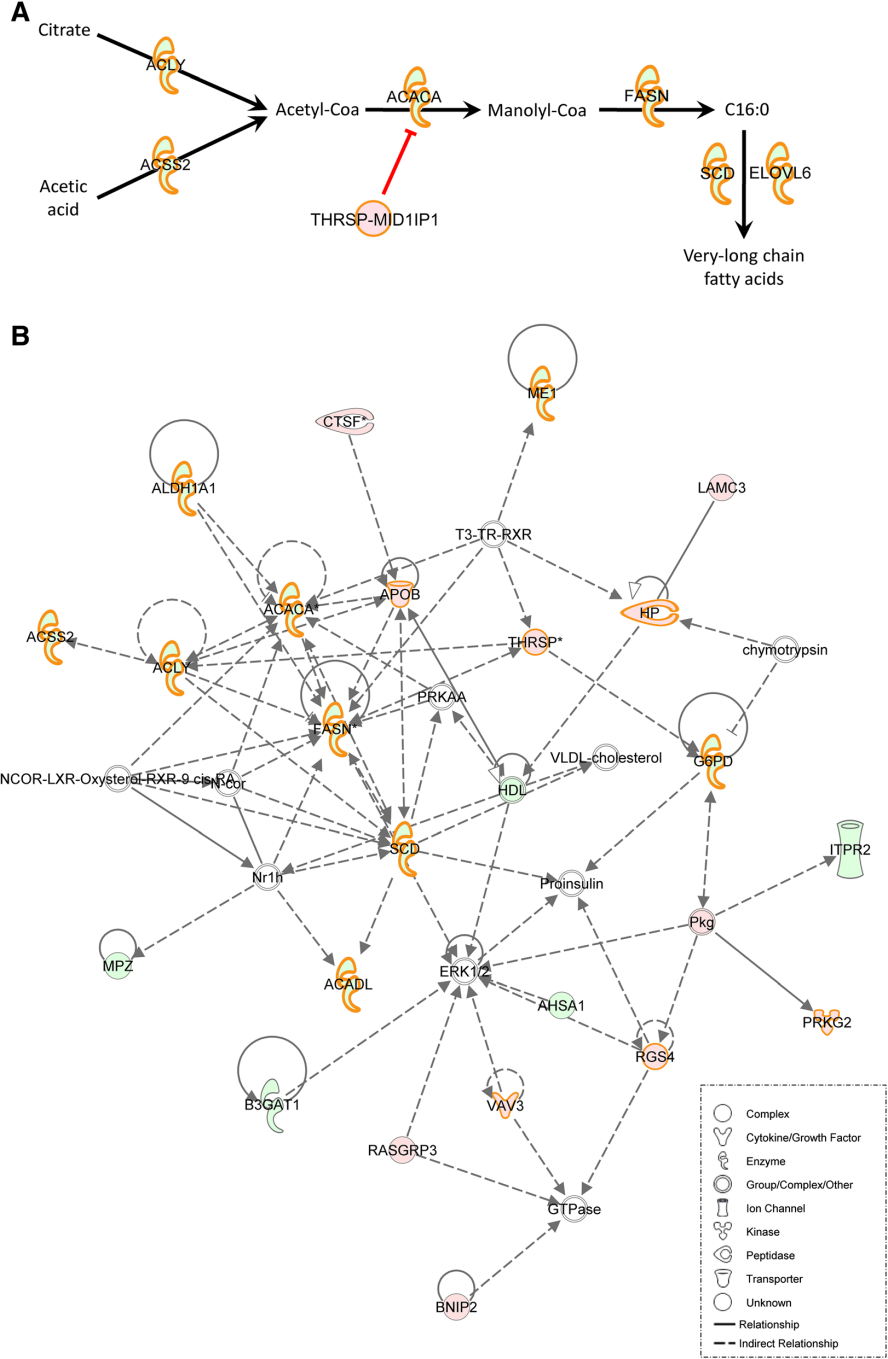
**Networks showing metabolic pathways differentially modulated between H and L groups. A**: Diagram of the different expression between H and L groups of the main genes affecting *de novo* FA synthesis pathway. **B**: Global IPA network of genes associated with lipid metabolism, nucleic acid metabolism and small molecule biochemistry. Biological association of 35 focus genes as a graphical representation of the molecular relationship (edges) between genes/gene products (nodes). The intensity of the node color indicates the degree of expression: (red) up-regulated and (green) down-regulated in the H group relative to the L group. The shape of nodes indicates the functional classes of the gene products. Genes highlighted in orange are those genes related to lipid metabolism.

To gain insight into the metabolic processes that differed between both groups, the list of 396 differentially expressed genes was analyzed using the core analysis function included in Ingenuity Pathways Analysis (IPA). The main biological functions identified were related to cancer, lipid concentration, synthesis of lipids, homeostasis of blood, and FA metabolism (Table 3). Interestingly, within the general representation of lipid metabolism, *de novo* FA synthesis pathway is remarkable where the most relevant genes showed lower expression in the H group. The ATP citrate lyase (ACLY) (fold difference = −2.07, p-value = 2.47 × 10^-04^) is the primary enzyme responsible for the synthesis of cytosolic acetyl-CoA from citrate and CoA in many tissues (Figure 3A) [35]. Although citrate is the main substrate for iniciating *de novo* lipogenesis in pigs, acetic acid is also used to produce cytosolic acetyl-CoA in species with extensive forestomach and hindgut fermentation such as rabbits, cattle, sheep and goats. The gene responsible for acetic acid conversion is the *acyl-CoA synthetase short-chain family member 2* (*ACSS2*) (fold difference = −2.12, p-value = 9.58 × 10^-05^), which was also less expressed in the H group, suggesting that cytosolic acetate may also contribute to increase lipogenesis in pig adipose tissue (Figure 3A). Both genes provide the substrate necessary for acetyl-CoA carboxylase alpha (ACACA) to iniciate *de novo* FA synthesis. The *ACACA* gene (fold difference = −2.67, p-value = 9.2 × 10^-07^) encodes the enzyme that catalyzes the carboxylation of acetyl-CoA to malonyl-CoA [36] (Figure 3A). This gene was previously reported as a candidate gene for a porcine quantitative trait *locus* (QTL) affecting the percentages of palmitoleic, stearic and vaccenic acids in chromosome 12 [36,37]. The product of ACACA is catalyzed by the *fatty acid synthase* (*FASN*) gene (fold difference = −9.3, p-value = 3.28 × 10^-16^) to synthesize palmitate (C16:0) in the presence of NADPH [38,39] (Figure 3A). These steps are important for the conversion of intermediate metabolites into FAs, thus contributing to the synthesis of cellular lipids and storage of fats. The lower expression levels of these genes may explain the minor content of C16:0 observed in L-group animals. The C16:0 FA produced in the cytosol is transferred to endoplasmic reticulum (ER) membranes, where the *ELOVL fatty acid elongase 6* (*ELOVL6*) gene (fold difference = −1.93, p-value = 7.2 × 10^-04^) and *stearoyl-CoA desaturase* (*SCD*) gene (fold difference = −7.87, p-value = 1.95 × 10^-06^) are sequentially involved to produce C18:1 [40] (Figure 3A). Some studies performed in our group proposed the *ELOVL6* gene as a candidate gene explaining a QTL in porcine chromosome 8, related to C16:0 and C16:1n-7 in the same animal material [41]. Despite no differences being observed between H and L groups of animals in backfat thickness (BFT) and IMF content, down-regulation of this gene has recently been associated with a higher fat content [42]. Moreover, the lower expression, in the H group, of genes related to glucose metabolism, such as *glucose-6-phosphate dehydrogenase* (*G6PD*) or *malic enzyme 1* (*ME1*), whose functions in glucose metabolism contribute to the initial steps of lipogenesis, is worth noting. The porcine *G6PD* gene (fold difference = −1.70, p-value = 8.2 × 10^-03^) product is a cytosolic enzyme responsible for the first step of a chemical pathway that converts glucose to ribose-5-phosphate. On the other hand, the porcine *ME1* gene (fold difference = −2.12, p-value = 2.1 × 10^-04^) encodes a tetrameric NADP-dependent enzyme that catalyzes the reversible oxidative decarboxylation of L-malate to pyruvate [43]. This gene was previously reported as a candidate gene for a porcine QTL affecting fat deposition in chromosome 1 [43]. Both genes contribute to produce pyruvate, which is transported and metabolized in the mitochondria to produce citrate. This citrate is the substrate of the differentially expressed *ACLY* gene and the initial molecule of pig lipogenesis. This complete metabolic pathway, from glucose metabolism to *de novo* lipogenesis, is well represented in the first IPA-generated network identified as “Lipid metabolism, Nucleic acid metabolism, Small molecule biochemistry” (score 36, focus molecules 23) (Figure 3B) (Additional file 7: Table S6). In clear consistency with the generated networks, the most representative canonical pathway significantly modulated between groups was the LXR/RXR activation (p-value = 6.19 × 10^-10^) (Table 4), which regulates the whole *de novo* FA synthesis pathway (Figure 3). Other canonical pathways differentially modulated between groups were ethanol degradation II, noradrenaline and adrenaline degradation, acute phase response signaling and the super-pathway of serine and glycine biosynthesis I (Table 4). On the other hand, the lower representation of *de novo* FA synthesis observed in the H group was validated by the higher expression of the *thyroid hormone responsive* (*THRSP*) gene (fold difference = 3.46, p-value = 3.36 × 10^-06^). This gene is abundantly expressed in lipogenic tissues and plays an important role in the biosynthesis of triglycerides with a medium-length FA chain and in modulating lipogenesis [44]. The co-expression of this gene with *MID1 interacting protein 1* (*MID1IP1*) leads to form a heterodimer between these proteins, which in turn inhibits the *MID1IP1* function of up-regulating the ACACA enzyme [44] (Figure 3A). All together, these results show that the main difference observed between the two groups of animals is the differential expression of genes implicated in *de novo* FA synthesis pathway. Results obtained may be explained by the adaptation of Iberian breeds (similar to L group) to sparse food availability, whereas Landrace pigs (similar to H group) are more sensitive to such variations due to this breed has been selected for fast growth. In support of our results, several key genes involved in glucose metabolism and *de novo* FA synthesis had been previously reported as differentially expressed between pigs with a lean phenotype (Landrace) and pigs with an obese phenotype (Rongchang pig) in adipose tissue [45]. Hence, differences in rates of tissue lipid accumulation between leaner pigs, as compared to fatter pigs, are attributable to genetic factors resulting in different patterns of expression of anabolic and oxidative lipid metabolism genes.

**Table 3:** Top five biological functions significantly modulated in backfat adipose tissue when comparing H vs. L animals

**Function**	**Genes**	**p-Value**
**Cancer**	*ACADL, ACAN, ACBD4, ACE2, ACLY, ACTG2, ACVR1C, ADH1A, AFAP1L1, AHSG, ALB, ALDH1A1, ALDOC, ANXA4, AP3M2, APAF1, APOB, AQPEP, ATP5J2, AZGP1, BCHE, BCL10, BMPER, BNIP2, C10orf116, C19orf53, C2orf40, C8A, CA3, CAPN6, CAPZA2, CCBP2, CCL21, CCT6A, CD1E, CD300LG, CENPF, CES1, CHST13, CLCA2, CLEC2D, CLIC5, CLK1, CMPK2, CNN1, COL11A1, COL15A1, COL5A1, COL8A2, COMT, CPXM2, CRABP1, CRABP2, CTCFL, CTNNAL1, CTSF, CXCL1…*	3.36E-07
**Concentration of lipids**	*ACACA, ACADL, ACLY, AHSG, ALB, ALDH1A1, APAF1, APOB, APOC3, CD4, CES1, CIDEC, COMT, CTDNEP1, CYP2E1, DHCR24, FASN, FFAR4, GC, GNAT1, HIF1AN, HP, MOGAT2, PHGDH, PLP1, PON1, PON2, PON3, RBP1, RDH16, RGS4, SCD, SNCA, STEAP4, TGFBR2, THEM5, THRSP, UGT8, VAV3*	1.22E-06
**Synthesis of lipids**	*ACACA, ACADL, ACLY, ACSS2, ALB, ALDH1A1, APOB, APOC3, C1QTNF3, CD4, CXCL1, CYP2E1, DHCR24, ESRRG, FASN, G6PD, KDR, KIT, LSS, MOGAT2, NFATC2, PLP1, PMVK, PON1, PON2, PRKG2, RBP1, RDH16, RDH5, SCD, SLC6A6, SNCA, THRSP, UGT8*	4.85E-06
**Homeostasis of blood**	*AHSG, APOC3, APOH, CIDEC, COMT, CYP2E1, ESRRG, MUT, VAV3*	9.46E-06
**Fatty acid metabolism**	*AACS, ACACA, ACADL, ACADSB, ACLY, ACSS2, ALB, APOB, APOC3, APOH, CD4, CXCL1, CYP2E1, EPHX1, FASN, GC, GM2A, KIT, ME1, NFATC2, PHGDH, PLP1, SCD, SLC36A2, SLC38A2, SLCO1A2, SNCA, UGT8*	1.32E-05

**Table 4:** Top five canonical pathways significantly modulated in backfat adipose tissue when comparing H vs. L animals

**Ingenuity canonical pathway**	**Genes**	**p-Value**
**LXR/RXR activation**	*SCD, APOB, APOH, AHSG, PCYOX1, PON1, ALB, LYZ, APOC3, FASN, ACACA, S100A8, FGA, GC, PON3, TNFRSF11B*	6.19E-10
**Ethanol Degradation II**	*ALDH4A1, ADH1A, ALDH1A1, ACSS2, PECR, ADHFE1*	2.52E-05
**Noradrenaline and adrenaline degradation**	*ALDH4A1, ADH1A, ALDH1A1, COMT, PECR, ADHFE1*	3.58E-05
**Acute phase response signaling**	*ALB, HP, RBP7, APOH, AHSG, CRABP2, FGB, FGA, RBP1, FGG, CRABP1, TNFRSF11B*	5.95E-05
**Superpathway of serine and glycine biosynthesis I**	*PSPH, PHGDH, SHMT2*	1.14E-04

### Differentially modulated pathways and pig lipid metabolism

**Figure 4:**
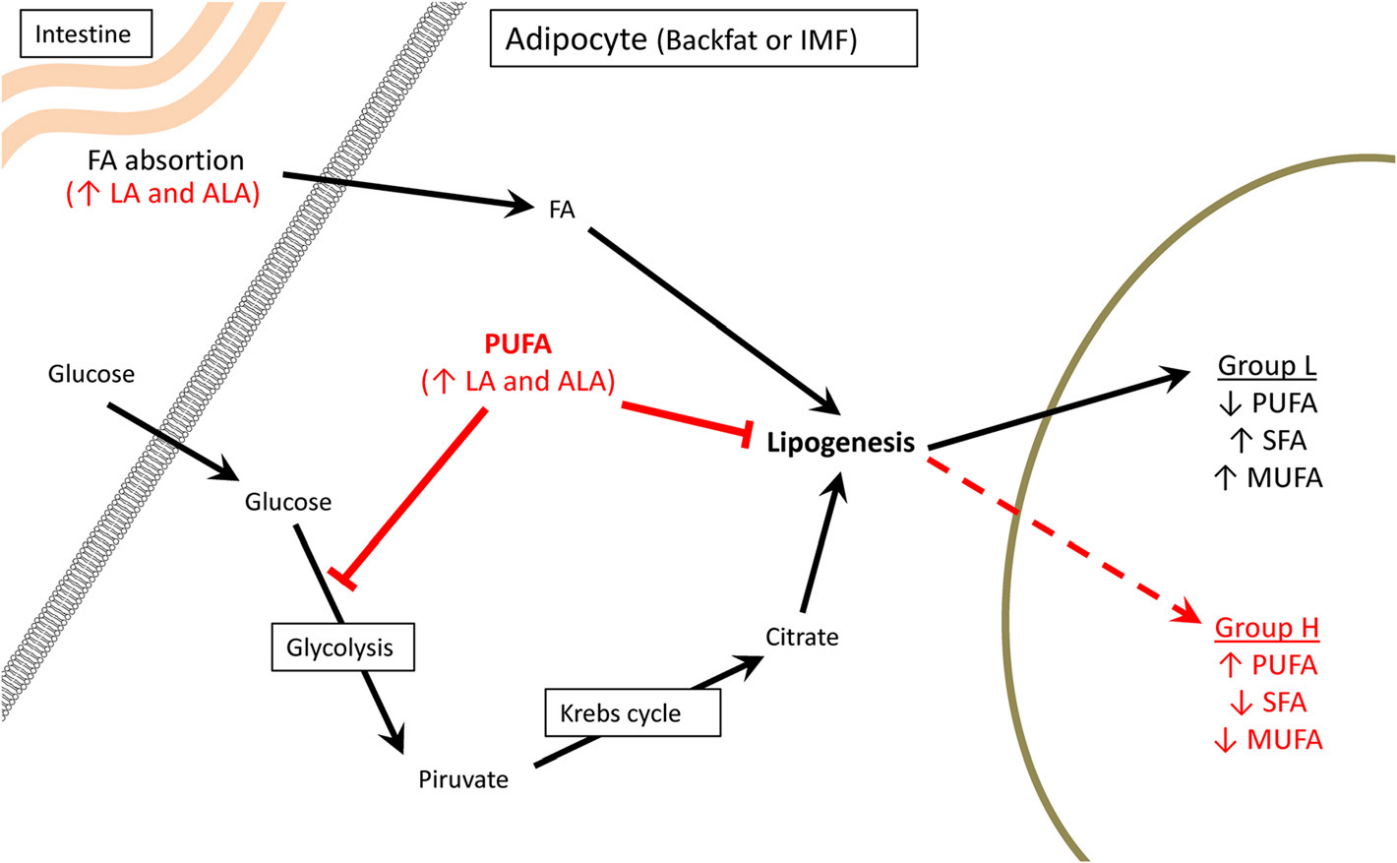
**Diagram representing the hypothetical causes of the differences in intramuscular and backfat FA composition.** Adipocytes incorporate FA from intestine absorption and glucose. Both follow different metabolic pathways that allow FA synthesis by lipogenesis (black arrows). Nevertheless, higher absorption, transport or storage of FA (mainly LA and ALA) produced an inhibitory effect (red lines) on the glucose metabolism and lipogenesis, reducing the content of MUFA and SFA and increasing PUFA content. FA: fatty acids, LA: linoleic acid and ALA: α-linolenic acid.

Previous studies suggested that differences in FA composition between Iberian and Landrace pigs may be an indirect consequence of differences in *de novo* lipid synthesis [26,46]. In this sense, animals of the H group will produce a lower proportion of SFA and MUFA, which must cause a decrease on triacylglycerides synthesis and the storage pathway. However, none of these pathways showed a down-representation in animals from the H group. Additionally, a lower lipid production in H-group animals would affect the IMF and BFT long-term, but no significant differences were observed between animals of the H and L groups. An alternative model for explaining this difference in IMF FA composition was previously suggested by [22], considering that the higher content of PUFA (mainly linoleic and α-linolenic acids) in H-group animals may be caused by a greater absorption, transport, storage or a lower degradation of essential FAs. Consequently, this higher amount of PUFA inhibits lipogenic activity in animals of the H group (Figure 4). This hypothesis would be a plausible explanation of the differences in FA accumulation observed between groups in adipose tissue and muscle, despite all animals having been fed the same diet.

FFAs derived from adipose tissue and VLDL-associated triglycerides derived from the liver are important sources of the FA supply to muscle, playing an important role in determining the intramuscular FA composition [15,23]. In fact, high, positive phenotypic correlations between adipose tissue and muscle FA composition were found for C14:0 (r_C14:0_ = 0.59, p-value = 1.14 × 10^-14^), C16:0 (r_C16:0_ = 0.72, p-value = 2.2 × 10^-16^) and C17:0 (r_C17:0_ = 0.65, p-value = 2.2 × 10^-16^), and moderate, positive phenotypic correlations were found for C16:1 n-7 (r_C16:1 n-7_ = 0.50, p-value = 3.3 × 10^-10^), C16:1 n-9 (r_C16:1 n-9_ = 0.47, p-value = 3.9 × 10^-09^), C18:0 (r_C16:0_ = 0.43, p-value = 9.8 × 10^-08^) and C18:1 n-9 (r_C18:1 n-9_ = 0.40, p-value = 9.2 × 10^-07^) in our animal material (Muñoz *et al*. (2013), submitted). In a previous study [22] that analyzed the liver transcriptome in the same groups of animals, it was suggested that a higher PUFA content observed in the H group induced a greater stimulation of both peroxisomal and mitochondrial β-oxidation and reduced triglyceride and cholesterol synthesis. This increase of FA oxidation observed in the liver of animals of the H group, jointly with ketone body production, is a “glucose sparing” mechanism of regulation in fasting conditions [32] which the animals were in at slaughter. In adipose tissue, the fasting condition induces the lipolysis of triglycerides storage and the blood transport of FFAs bound to albumin (ALB) (fold difference = 3.05, p-value = 1.23 × 10^-07^) to organs such as the heart and skeletal muscle to fulfill their energy requirements [47]. Previous studies performed in 3T3-L1 pre-adipocytes demonstrated that over-expression of *ALB* stimulates long-chain FAs uptake by direct interaction with adipose cells and suggested that this stimulatory effect may be a general phenomenon in other types of cells [48]. Hence, data obtained may explain the greater uptake of FAs into hepatocytes and their degradation in the β-oxidation pathway in the liver of the H-group animals. The negative effect that dietary PUFA causes on *de novo* FA synthesis [33,49,50] is well known, and this effect was also observed in our data. The down-regulation of this pathway in the group with the higher content of PUFA (that is, the H group) may be caused by the inhibitory effect of *n-3* and *n-6* PUFA on the expression of *receptor subfamily 1, group H, member 3* (*NR1H3*) [50]. The *NR1H3* gene, also called *liver X receptor α* (LXRα), is a nuclear receptor which is highly expressed in adipose tissue. Studies performed in *NR1H3*^(−/−)^ mice showed a decrease on *de novo* FA synthesis, due to the down-regulation of *SREBF1* and its target genes [51] (Figure 3). Other studies confirmed that *ChREBP* is a key transcriptional regulator for the coordinated inhibition of glycolytic and lipogenic genes by PUFA [52]. PUFA also suppresses the *ChREBP* gene function in a LXR-dependent manner, increasing its mRNA decay and altering ChREBP protein translocation from the cytosol to the nucleus [33]. In addition, we cannot rule out a direct inhibition of *SCD* expression by PUFA [53] in animals of the H group. The repression of *SCD* increases the intracellular pool of saturated fatty acyl-CoAs inhibiting the ACACA enzyme and *de novo* lipogenesis and activating the *carnitine palmitoyltransferase 1* (*CPT1*) gene, which is responsible for the rate-limiting step in the importing and oxidation of FAs into the mitochondria [54]. Thus, altogether, our results are in agreement with a functional and anatomical separation of *de novo* lipid synthesis and β-oxidation in the porcine adipose tissue and liver, respectively. This suggests a tightly coordinated process among different hormones (peptides and/or lipids), transcription factors and nuclear receptors to avoid the simultaneous activation of antagonistic pathways. However, there is great controversy in explaining the relevance of β-oxidation in porcine adipose tissue. PPARA is considered to be the main transcription factor controlling FA oxidation. There are some studies that described a greater expression of pig *PPARA* in adipose tissue than in the liver, suggesting that adipose tissue could oxidize FAs to any extent [55]. In contrast, other authors did not find *PPARA* expression in porcine adipose tissues [56]. Consistent with this, we found higher levels of *PPARA* expression in the liver, as compared to adipose tissue, which suggests an important role of the liver in porcine β-oxidation.

Different studies have determined the importance of several adipose tissue-derived hormones in the regulation of systemic carbohydrate and lipid homeostasis [57,58]. Communication between adipose tissue and distant organs has been previously described through the lipokine palmitoleate (C16:1 n-7), which strongly stimulates muscle insulin action while it suppresses hepatosteatosis [15]. Studies performed *in vivo* in humans showed a clear increase of *SREBF1c* caused by insulin in muscle and, consequently, the induction of key lipogenic enzymes [59,60]. The mean comparison of C16:1 n-7 FA composition between the L and H groups showed suggestive differences in both muscle (Additional file 1: Table S1) and adipose tissue (data not shown). Hence, different levels of C16:1 n-7 may determine a differential systemic regulation that may explain the phenotypic variations observed between groups. Lipogenesis in muscle is produced by the small proportion of adipocytes in this tissue. Therefore, their lipogenic activity is lower in comparison with other extra-muscular adipose tissues [61]. Despite the lower rate of lipogenesis observed in muscle, *de novo* FA synthesis directly contributes to the *in situ* intramuscular FA composition [61]. In this sense, the study of muscle transcriptome in pigs, together with liver and adipose tissue transcriptomes, would be important in order to obtain a complete view of FA metabolism.

Finally, other adipose tissue-derived hormones such as leptin or adiponectin cannot be discarded, as well as other interesting genes not annotated in the current pig genome assembly. The differentially expressed genes identified seem to be relevant in controlling the overall FA composition in adipose tissue and muscle, and they should be considered as candidate genes for meat quality traits in pigs. The knowledge of these genes and their regulatory networks may help in the design of new strategies for improving pork meat quality by increasing the MUFA/SFA and n-3/n-6 PUFA ratios [2]. The maintenance of these ratios is essential in order to reduce the imbalanced FA intake of today’s consumers and to avoid several diseases, including cancers and coronary heart disease. The great similarities between pigs and humans in body size and other physiological/anatomical features convert the pig into an excellent biomedical model for human disease. Hence, analysis of the porcine backfat transcriptome showed the role of several genes in regulating the lipid metabolism not only in pigs but also in humans, due to the metabolic similarities between both species. Additionally, several lipid-related diseases affected both human and pig (obesity, including diabetes, metabolic syndrome or other lipid-related diseases), therefore, data generated in this study can be used to identify polymorphism with a major effect on these diseases.

## Conclusions

In this study, we provide a global view of the adipose tissue (backfat) transcriptome of six pigs and extensive new knowledge about transposable elements, new putative protein-coding genes and the expression levels of known genes in adipose tissue. Animals were classified into two groups according to their intramuscular FA composition, and 396 genes were found to be differentially expressed between groups. These genes belong to molecular functions and gene networks related to lipid and FA metabolism. Pathway analysis showed a different modulation of lipogenesis between phenotypically extreme animals, probably caused by differences in PUFA levels (mainly linolenic and α-linolenic). Finally, the crucial role of IMF FA composition in the technological and the nutritional and organoleptic quality of pork meat is well-known. Hence, this study will allow for the identification of candidate genes and gene networks for FA composition traits which may help in the design of better selection strategies to improve porcine meat quality traits.

## Methods

### Animal material

The IBMAP cross was originated by crossing three Iberian (Guadyerbas line) boars with 31 Landrace sows [62]. Animals used in this study belong to a backcross (BC1_LD) generated by crossing five F1 (Iberian × Landrace) boars with 26 Landrace sows and producing 144 backcrossed animals. All pigs were raised in a normal intensive system and feeding was *ad libitum* with a cereal-based commercial diet. Pigs were slaughtered at an average age of 179.8 days ± 2.6 days following national and institutional guidelines for the ethical use and treatment of animals in experiments. Samples of adipose tissue (backfat) were collected at the slaughterhouse, snap-frozen in liquid nitrogen and stored at −80°C until analyzed. A total of 48 traits related to growth, carcass quality and intramuscular FA composition were measured. In Ramayo-Caldas *et al.* (2012), the phenotypic information from twenty-six of the total traits was used to classify BC1_LD animals into two groups (H and L) according to the first component of a PCA [22]. A total of six animals was selected for the study, considering pedigree information representing the parental genetic diversity, and that only females were retained for RNA sequencing (three per group). Phenotypic mean comparison between groups was performed using a linear model implemented in R.

### RNA isolation, library preparation and sequencing

Total RNA was isolated from backfat using the *RiboPure*™ *Isolation of High Quality Total RNA* (*Ambion®; Austin, TX*) following the manufacturer’s recommendations. RNA was quantified using the *Nano-Drop ND-1000 spectrophotometer* (*NanoDrop products;* Wilmington, USA) and checked for purity and integrity in a *Bioanalyser-2100* (*Agilent Technologies, Inc.,* Santa Clara CA, USA). For each sample, one paired-end library with an approximately 300-bp insert size was prepared using TruSeq RNA Sample Prep Kit v2 (*Illumina, Inc.*; San Diego CA, USA). To discriminate among samples, libraries were labeled by barcoding and pooled to be run in different lanes. Libraries were sequenced, in CNAG (Centro Nacional de Análisis Genómico), on an Illumina HiSeq2000 instrument (*Illumina, Inc.*; San Diego CA, USA) that generated paired-end reads of 75 nucleotides. More than 236 million reads were generated in this study.

### Mapping, assembling and annotation of reads

In order to map all reads generated, the software TopHat v2.0.1 [63,64] was employed, using as reference version 10.2 of the pig genome (Sscrofa10.2) and the annotation database Ensembl Genes 67 [http://www.ensembl.org/info/data/ftp/index.html]. Tophat was used with an expected mean inner-distance of 160-bp between paired-end reads. Quality control and reads statistics were determined with FASTQC [http://www.bioinformatics.babraham.ac.uk/projects/fastqc/]. Transcripts were assembled and quantified by Cufflinks v2.0.2 [64,65] with a minimum alignment count per *locus* of 20. Additionally, for counting the number of reads mapping to exons, introns and intergenic positions, the intersectBED tool from BEDtools was used [66].

### Orthology detection and transposable element analysis

Intergenic-expressed regions, according to the current pig genome assembly (Sscrofa10.2), were extracted with Cuffcompare [65] and custom Python and R scripts. Putative coding transcripts were identified with Augustus [29], providing exon boundaries and allowing for complete protein translation. Functional annotation was performed by using BLASTP option from BLAST2GO, with the following parameters: *E-*value hit filter 1.00E-6, annotation cutoff 55, gene ontology (GO) weight 5 and HSP-hit coverage cutoff 0 [67]. Additionally, an InterProScan tool implemented in the BLAST2GO software and the ANNEX data set was employed to refine the functional annotations. GO terms were summarized according to the three principal GO categories: cellular component, biological process and molecular functions. Enzyme mapping of annotated sequences was performed using direct GO to enzyme mapping and used to query the Kyoto Encyclopedia of Genes and Genomes (KEGG) to define the main metabolic pathways involved [68,69].

Furthermore, the software RepeatMasker (http://www.repeatmasker.org/) version open-3.30 was employed with the ‘quick search’ option and ‘pig’ species, in order to indentify repetitive and transposable elements in the adipose tissue transcriptome. The Search Engine used was NCBI/RMBLAST with the complete rm-20120418 database.

### Gene expression quantification and correlation analysis with expression microarrays

Qualimap v0.5 software was employed to count the number of reads mapped for each gene, and the total number of counts was considered as expression values [70]. Pearson correlations between the mean expression values of each group were calculated using the *cor.test* function of R. Five of the animals sequenced were also assayed with high-density oligonucleotide microarray chips (*GeneChip*® *Porcine*) from *Affymetrix* (Santa Clara, CA), containing a total of 23,937 probe sets (23,256 transcripts), representing 20,201 *Sus scrofa* genes. Microarrays were hybridized and scanned at the *Institut de Recerca Hospital Universitari Vall d’Hebron* (Barcelona, Spain) following *Affymetrix* standard protocols. The Gene-Chip Operating Software (GCOS) was used to generate expression data, and probes were normalized and adjusted for background noises with the GCRMA R package [71]. All probes correspond to a total of 7,885 Ensembl gene IDs expressed in backfat, and these genes were used to estimate the Spearman correlation between the log2 expression values of genes analyzed by RNA-Seq and microarrays.

### Differential gene expression analysis

The DESeq R package was employed to detect genes differentially expressed between groups [72]. DESeq mediates a negative binomial distribution by modeling the biological and technical variance for testing DE genes in two experimental conditions. DESeq uses the unambiguous table of counts per gene obtained from QualiMap software using the *comp-counts* option as the input file [70]. Before the analysis, some exploratory tests were performed to validate both the good data quality and the variance estimation. Per-gene estimates of the base variance against the base levels showed that the fit (red line) followed the single-gene estimates well (Additional file 8: Figure S3). The *residualEcdfPlot* function used to check the uniformity of the cumulative probabilities revealed a similar curve pattern of the empirical cumulative density functions (ECDF) in both groups. Data were filtered by a minimum mean of 20 reads mapped per gene and only those genes with a fold difference between groups higher than 1.2-fold or lower than −1.2-fold were retained. Fold differences were calculated referring to the group of animals with the lower expression. Genes with a positive fold difference indicate that they are highly expressed in the H group, whereas genes with negative fold difference values represent that they are highly expressed in the L group. Then, the R package q-value [73] was employed to calculate the false-discovery rate, and genes with a p-value ≤ 0.01 (which is equivalent to a q-value ≤ 0.1) were retained in both classifications.

### Gene functional classification, network and canonical pathways analyses

A bioinformatics approach was used to elucidate the biological importance of differentially expressed genes in the adipose tissue transcriptome. Ingenuity Pathway Analysis software (IPA; Ingenuity Systems, http://www.ingenuity.com) was applied to identify functions and pathways represented and for generating biological networks. The IPA program consists of the Ingenuity Pathway Knowledge Base (IPKB), which is derived from known functions and interactions of genes published in the literature. IPA presents the top canonical pathways associated with the uploaded data with a p-value calculated using the right-tailed Fisher’s exact test. Functional analysis was used to identify the biological functions that are differentially represented between both groups (H and L). Networks were algorithmically generated based on their connectivity, with a score representing the log probability of a particular network being found by random chance. Direct and indirect biological relationships between molecules (nodes) were represented as continuous and discontinuous lines, respectively. All lines are supported by at least one reference from the literature, from a textbook, or from canonical information stored in the Ingenuity Pathways Knowledge Base. The intensity of the node color indicates the degree of up-(red) or down-(green) regulation of the H versus L group.

### Data availability

The full data sets have been submitted to Gene Expression Omnibus (GEO) under accession GSE52012 and at NCBI Sequence Read Archive (SRA) under Accession SRP031783, Bioproject: PRJNA223356.

## Competing interests

The authors declare that they have no competing interests.

## Authors’ contributions

JC, JMF and MB conceived and designed the experiment. JMF was the principal investigator of the project. JC, YRC and APO performed the RNA-Seq data analysis. JC and JE performed the pathways analysis. JC, MB and AC performed the RNA isolation. EA, RP, JE and JMF collected the samples. JC, JMF and MB drafted the manuscript. All authors read and approved the final manuscript.

## Supplementary Material

Additional file 1: Table S1 Mean (±SEM) comparison between H and L groups of the intramuscular fatty acid composition traits. **Table S2.** Cufflinks transcript assembly (TA) statistics for each sample. Click here for file

Additional file 2: Table S3 Gene ontology (GO) of the novel predicted proteins in adipose tissue transcriptome. Click here for file

Additional file 3: Table S4 Description of the repetitive elements identified in the pig adipose tissue transcriptome. Click here for file

Additional file 4: Figure S1 Distribution of gene expression levels in both H (High) and L (Low) groups. Click here for file

Additional file 5: Figure S2 Correlation between expression values of RNA-Seq and Affymetrix microarray. X-axis values are the log2 of expression quantified with Affymetrix microarray technology and y-axis are values of log2 (counts). Click here for file

Additional file 6: Table S5 Differential-expressed genes between H and L groups with a fold difference ≥ 1.2 and a p-value ≤ 0.01. Click here for file

Additional file 7: Table S6 Genetic networks generated from the differential expressed genes between H and L animals. Click here for file

Additional file 8: Figure S3 Per-gene estimates of the base variance against the base level. The red line represents the fit variance. X-axis is the log10 of the base mean and y-axis values are the log10 of the base variance. Click here for file
