# Supplementary material for: Analysis of porcine adipose tissue transcriptome reveals differences in de novo fatty acid synthesis in pigs with divergent muscle fatty acid composition
Source: BMC Genomics. 2013 Dec 1;14:843. doi: 10.1186/1471-2164-14-843 (PMC3879068; doi:10.1186/1471-2164-14-843)
Supplement: Additional file 1: Table S1 — Mean (±SEM) comparison between H and L groups of the intramuscular fatty acid composition traits. Table S2. Cufflinks transcript assembly (TA) statistics for each sample. [file 1471-2164-14-843-S1.docx]

| **Carcass quality** | **Group H** | **Group L** | **Significance** | **p-value** |
| --- | --- | --- | --- | --- |
| Intramuscular fat (IMF) | 2.27 ± 0.63 | 1.76 ± 0.75 | NS | 0.41796 |
| Carcass weight (CW) | 64.10 ± 16.13 | 67.50 ± 8.55 | NS | 0.76321 |
| Ham weight (HW) | 17.72 ± 3.15 | 17.18 ± 0.87 | NS | 0.78914 |
| Shoulder weight (SW) | 6.04 ± 1.04 | 5.67 ± 0.34 | NS | 0.59235 |
| Backfat thickness (BFT) | 13.33 ± 2.52 | 18 ± 5.20 | NS | 0.23410 |
| **Fatty acids** | | | | |
| ***Saturated FA*** | | | | |
| Myristic acid (C14:0) | 1.09 ± 0.18 | 1.26 ± 0.08 | NS | 0.21009 |
| Palmitic acid (C16:0) | 21.45 ± 0.70 | 24.01 ± 0.65 | ** | 0.0095 |
| Heptadecenoic acid (C17:0) | 0.38 ± 0.05 | 0.21 ± 0.04 | * | 0.0101 |
| Stearic acid (C18:0) | 14.00 ± 1.11 | 13.90 ± 1.02 | NS | 0.91878 |
| Arachidic acid (C20:0) | 0.33 ± 0.03 | 0.26 ± 0.05 | NS | 0.12730 |
| ***Monounsaturated FA*** | | | | |
| Palmitoleic acid (C16:1 n-7) | 2.25 ± 0.43 | 2.95 ± 0.20 | · | 0.06403 |
| Heptadecenoic acid (C17:1) | 0.36 ± 0.10 | 0.26 ± 0.03 | NS | 0.17130 |
| Oleic acid (C18:1 n-9) | 35.16 ± 3.94 | 43.17 ± 1.26 | * | 0.02840 |
| Octadecenoic acid (C18:1 n-7) | 3.86 ± 0.20 | 4.10 ± 0.19 | NS | 0.21116 |
| Eicosenoic acid (C20:1 n-9) | 0.88 ± 0.18 | 0.80 ± 0.02 | NS | 0.47216 |
| ***Polyunsaturated FA*** | | | | |
| Linoleic acid (C18:2 n-6) | 13.64 ± 1.99 | 6.84 ± 0.63 | ** | 0.00485 |
| α-Linolenic acid (C18:3 n-3) | 1.43 ± 0.44 | 0.52 ± 0.04 | * | 0.02448 |
| Eicosadienoic acid (C20:2 n-6) | 0.51 ± 0.12 | 0.37 ± 0.06 | NS | 0.13036 |
| Eicosatrienoic acid (C20:3 n-6) | 0.50 ± 0.21 | 0.21 ± 0.01 | * | 0.04215 |
| Arachidonic acid (C20:4 n-6) | 3.46 ± 1.54 | 0.74 ± 0.23 | * | 0.03899 |
| ***Metabolic ratios*** | | | | |
| Average Chain Length (ACL) | 17.46 ± 0.01 | 17.37 ± 0.01 | *** | 0.000678 |
| Saturated FA (SFA) | 37.23 ± 0.38 | 39.64 ± 1.17 | * | 0.02783 |
| Monounsaturated FA (MUFA) | 42.89 ± 3.64 | 51.63 ± 1.46 | * | 0.01810 |
| Polyunsaturated FA (PUFA) | 19.54 ± 3.95 | 8.61 ± 0.80 | ** | 0.00929 |
| PUFA(n-3)/PUFA(n-6) | 0.08 ± 0.02 | 0.07 ± 0.01 | NS | 0.32977 |
| Peroxidability index (PI) | 33.92 ± 8.78 | 13.51 ± 1.40 | * | 0.01644 |
| Double-bond index (DBI) | 0.48 ± 0.11 | 0.19 ± 0.02 | * | 0.01324 |
| Unsaturated index (UI) | 0.90 ± 0.08 | 0.71 ± 0.02 | * | 0.01319 |

**Table S1. Mean (±SEM)** **comparison between H and L groups of the intramuscular fatty acid composition traits.**

NS: p-value > 0.1, · p-value > 0.05, * p-value < 0.05, **p-value < 0.01, ***p-value <0.001

**Table S2. Cufflinks transcript assembly (TA) statistics for each sample**

| **Animals** | **BC1** | | **BC2** | | **BC3** | | **BC4** | | **BC5** | | **BC6** | |
| --- | --- | --- | --- | --- | --- | --- | --- | --- | --- | --- | --- | --- |
|  | **TA** | **%** | **TA** | **%** | **TA** | **%** | **TA** | **%** | **TA** | **%** | **TA** | **%** |
| **=** | 25,919 | 47 | 25,858 | 45 | 25,991 | 42 | 25,919 | 45 | 25,958 | 43 | 25,905 | 45 |
| **c** | 4 | 0 | 4 | 0 | 5 | 0 | 3 | 0 | 3 | 0 | 3 | 0 |
| **e** | 1,722 | 3 | 1,651 | 3 | 2,063 | 3 | 1,742 | 3 | 2,191 | 4 | 2,018 | 4 |
| **i** | 3,865 | 7 | 4,514 | 8 | 7,185 | 12 | 4,717 | 8 | 6,773 | 11 | 5,091 | 9 |
| **j** | 11,404 | 21 | 11,436 | 20 | 12,072 | 20 | 11,526 | 20 | 11,557 | 19 | 11,800 | 21 |
| **o** | 763 | 1 | 865 | 2 | 810 | 1 | 808 | 1 | 762 | 1 | 831 | 1 |
| **p** | 1,274 | 2 | 1,359 | 2 | 1,682 | 3 | 1,439 | 3 | 1,502 | 2 | 1,339 | 2 |
| **s** | 14 | 0 | 13 | 0 | 13 | 0 | 13 | 0 | 7 | 0 | 10 | 0 |
| **u** | 9,544 | 17 | 11,196 | 20 | 11,580 | 19 | 10,896 | 19 | 11,800 | 19 | 10,156 | 18 |
| **x** | 336 | 1 | 390 | 1 | 307 | 0 | 348 | 1 | 328 | 1 | 340 | 1 |
| **Total** | 54,845 | 100 | 57,286 | 100 | 61,708 | 100 | 57,411 | 100 | 60,881 | 100 | 57,493 | 100 |

BC1 to BC3 correspond to animals of the L group, while BC4 to BC6 correspond to animals of the H group. Class codes described by Cuffcompare: "=" Exactly equal to the reference annotation, "c " Contained in the reference annotation, "e" Possible pre-mRNA molecule, "i " An exon falling into an intron of the reference, "j " New isoforms, "o" Unknown, generic overlap with reference, "p" Possible polymerase run-on fragment, “s” An intron of the transfrag overlaps a reference intron on the opposite strand, "u" Unknown, intergenic transcript, “x” Exonic overlap with reference on the opposite strand.

**Table S3. Gene ontology (GO) of the novel predicted proteins in adipose tissue transcriptome.**

**Table S4. Description of the repetitive elements identified in the pig adipose tissue transcriptome.**

**Table S5. Differential-expressed genes between H and L groups with a fold difference ≥ 1.2 and a p-value ≤ 0.01.**

**Table S6. Genetic networks generated from the differential expressed genes between H and L animals.**

**Figure S1.** Distribution of gene expression levels in both H (High) and L (Low) groups.

**Figure S2.** Correlation between expression values of RNA-Seq and Affymetrix microarray. X-axis values are the log2 of expression quantified with Affymetrix microarray technology and y-axis are values of log2(counts).

**Figure S3.** Per-gene estimates of the base variance against the base level. The red line represents the fit variance. X-axis is the log10 of the base mean and y-axis values are the log10 of the base variance.
