# Supplementary figures and images for: Analysis of porcine adipose tissue transcriptome reveals differences in de novo fatty acid synthesis in pigs with divergent muscle fatty acid composition
Source: BMC Genomics. 2013 Dec 1;14:843. doi: 10.1186/1471-2164-14-843 (PMC3879068; doi:10.1186/1471-2164-14-843)

## L group

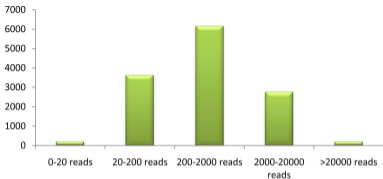

## H group

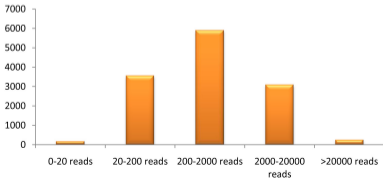

Supplement: Additional file 4: Figure S1 — Distribution of gene expression levels in both H (High) and L (Low) groups. [file 1471-2164-14-843-S4.pdf]

**BC2 ( $r = 0.67$ )**

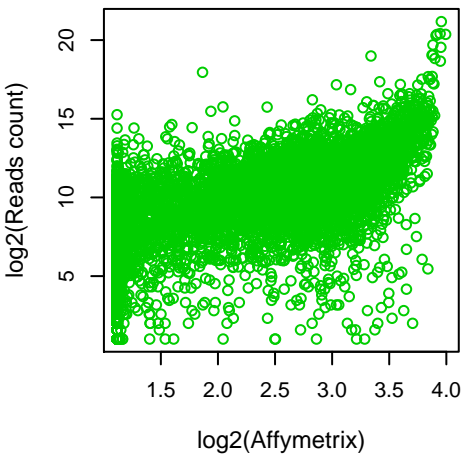

**BC3 ( $r = 0.67$ )**

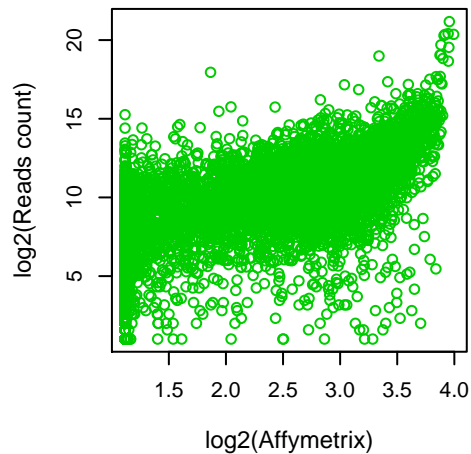

**BC4 ( $r = 0.68$ )**

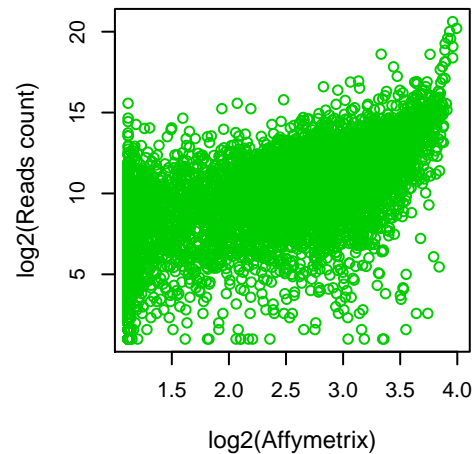

**BC5 ( $r = 0.67$ )**

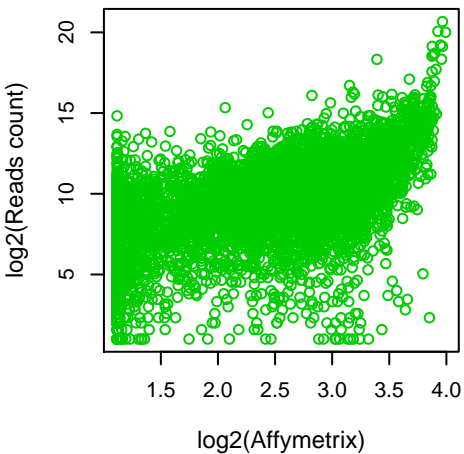

**BC6 ( $r = 0.65$ )**

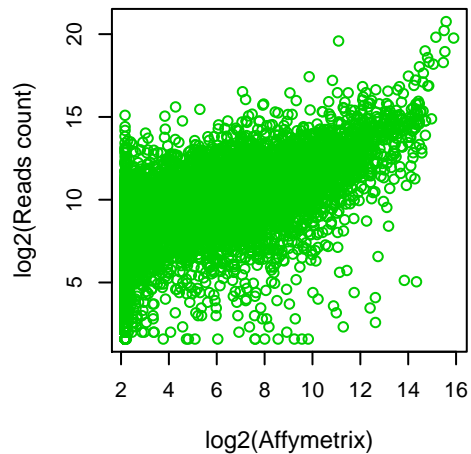

Supplement: Additional file 5: Figure S2 — Correlation between expression values of RNA-Seq and Affymetrix microarray. X-axis values are the log2 of expression quantified with Affymetrix microarray technology and y-axis are values of log2 (counts). [file 1471-2164-14-843-S5.pdf]

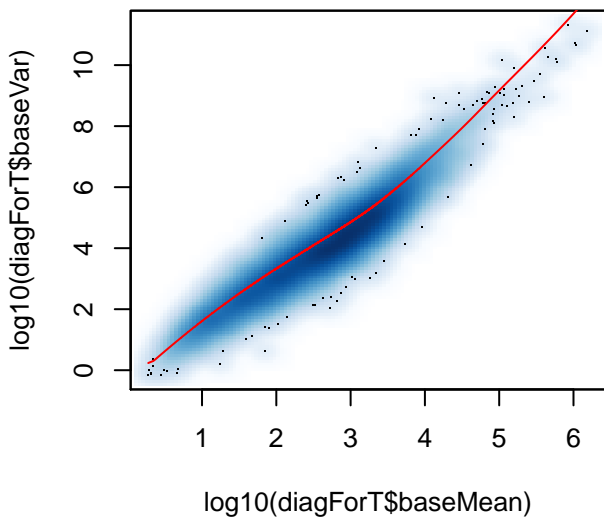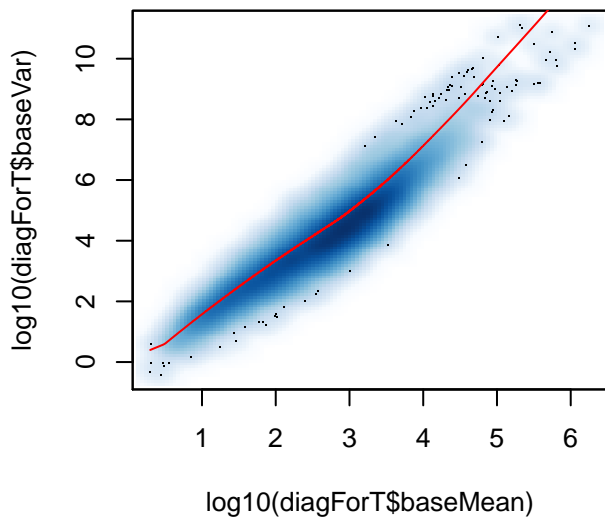

Supplement: Additional file 8: Figure S3 — Per-gene estimates of the base variance against the base level. The red line represents the fit variance. X-axis is the log10 of the base mean and y-axis values are the log10 of the base variance. [file 1471-2164-14-843-S8.pdf]
